# Developing a Conceptual Framework for Environmental Health Tracking in Victoria, Australia

**DOI:** 10.3390/ijerph16101748

**Published:** 2019-05-17

**Authors:** Benjamin Edokpolo, Nathalie Allaz-Barnett, Catherine Irwin, Jason Issa, Pete Curtis, Bronwyn Green, Ivan Hanigan, Martine Dennekamp

**Affiliations:** 1 Environmental Public Health Unit, Environment Protection Authority Victoria, 200 Victoria Street, Carlton, Victoria 3053, Australia; nathalie.allaz-barnett@epa.vic.gov.au (N.A.-B.); catherine.irwin@epa.vic.gov.au (C.I.); jason.issa@epa.vic.gov.au (J.I.); pete.curtis@epa.vic.gov.au (P.C.); bronwyn.green@epa.vic.gov.au (B.G.); martine.dennekamp@epa.vic.gov.au (M.D.); 2 School of Public Health, University Centre for Rural Health, The University of Sydney, Sydney 2006, Australia; ivan.hanigan@sydney.edu.au; 3 The Centre for Air pollution, energy and health Research (CAR), Woolcock Institute of Medical Research, Sydney 2006, Australia; 4 Centre for Research and Action in Public Health, University of Canberra, Canberra 2617, Australia; 5 School of Public Health and Preventive Medicine, Monash University, Melbourne 3004, Australia

**Keywords:** Environment Protection Authority Victoria, environmental health tracking, Driving Force-Pressure-Environmental Condition-Health Impact-Action (DPEHA) conceptual framework, environmental exposure, health effects

## Abstract

Victoria’s (Australia) Environment Protection Authority (EPA), the state’s environmental regulator, has recognized the need to develop an Environmental Health Tracking System (EHTS) to better understand environmental health relationships. To facilitate the process of developing an EHTS; a linkage-based conceptual framework was developed to link routinely collected environmental and health data to better understand environmental health relationships. This involved researching and drawing on knowledge from previous similar projects. While several conceptual frameworks have been used to organize data to support the development of an environmental health tracking system, Driving Force–Pressure–State–Exposure–Effect–Action (DPSEEA) was identified as the most broadly applied conceptual framework. Exposure and effects are two important components of DPSEEA, and currently, exposure data are not available for the EHTS. Therefore, DPSEEA was modified to the Driving Force–Pressure–Environmental Condition–Health Impact–Action (DPEHA) conceptual framework for the proposed Victorian EHTS as there is relevant data available for tracking. The potential application of DPEHA for environmental health tracking was demonstrated through case studies. DPEHA will be a useful tool to support the implementation of Victoria’s environmental health tracking system for providing timely and scientific evidence for EPA and other decision makers in developing and evaluating policies for protecting public health and the environment in Victoria.

## 1. Introduction

The introduction of chemicals, physical agents, and biological toxins to the environment, whether from accidental discharge, intentional release, or natural processes, can cause adverse changes in the environment. Identifying pollution sources, the causes, and links between changes in environmental conditions and adverse health effects will help prevent risk of harm to human health and the environment. Environmental health tracking (also called environmental health surveillance) is currently used by various organizations to better understand the impacts on human health from changes in environmental conditions and identify the most effective prioritising efforts and interventions. Some examples of international environmental health tracking systems are the World Health Organization (WHO) Europe Region Environmental Health Information System [1], United States Centers for Disease Control and Prevention Environmental Public Health Tracking Network [2], and Environmental Health Indicators New Zealand [3]. 

Environment Protection Authority (EPA) Victoria in Australia is the state’s environmental regulator, responsible for protecting the environment from pollution and waste in the state of Victoria. In March 2016, the Victorian Ministerial Advisory Committee (MAC) released a report on an independent inquiry into EPA Victoria [4]. One recommendation from the MAC report was the delivery of environmental health functions relevant to pollution and waste within the EPA. To strengthen its environmental health capability and better understand how environmental pollution from natural and man-made sources can affect public health, EPA Victoria is developing an environmental health tracking system. As defined by Thacker et al., environmental public health surveillance is “the ongoing collection, integration, analysis, and dissemination of data from environmental hazards monitoring, human exposure tracking, and health effect surveillance” [5]. The Environmental Health Tracking System (EHTS) will be used to provide timely and scientific information for EPA and other decision makers in Victoria for implementing and evaluating policies to effectively manage environmental and health-related issues in Victoria. It will allow for a better understanding of the possible associations between environmental exposures and health outcomes. This will then guide interventions aimed at improving public health for Victorians. 

Objectives for using an environmental health tracking system in Victoria are:

(1) To proactively investigate the short- and long-term temporal and spatial trends of environmental conditions and adverse health effects to help guide interventions and prevent harm to human health resulting from environmental pollution or waste.
(2) To have access to short- and long-term trends to respond to community concerns related to environmental public health issues.
(3) To make relevant environmental public health tracking data available to stakeholders (e.g., public, government departments, and researchers) on a spatial and temporal scale in an easy to understand format.
(4) To respond to emerging environmental health threats, clusters, and outbreaks by identifying populations at risk for adverse health effects.
(5) To investigate relationships between environmental contamination and health effects for specific cases.
(6) To develop routine tracking of adverse health effects on a small scale and identify areas warranting further investigation.
(7) To increase partnerships and collaborations between traditional environmental and health entities at federal, state, and local government levels by developing networks.

The successful implementation of an EHTS in Victoria will involve:

1. Investigating the feasibility of implementing EHTS in Victoria;
2. Developing a conceptual framework for the EHTS;
3. Developing and identifying environmental health indicators for the EHTS;
4. Developing a technical implementation plan for the system architecture of the EHTS;
5. Implementing the EHTS with available data; and
6. Evaluation of the effectiveness of the EHTS;

The outcome of the feasibility study indicated that an EHTS was feasible in Victoria, and this paper focused on step 2. The aim of this paper is to develop a conceptual framework for EPA Victoria’s EHTS. The proposed conceptual framework will support successful implementation of the EHTS in Victoria.

## 2. Strategy for Developing Environment Protection Authority (EPA)’s Environmental Health Tracking Conceptual Framework

In developing a conceptual framework for EPA’s environmental health tracking, a literature review and in-depth discussions with a wide range of experts via focused workshops were carried out. The expert workshops were organized by EPA Victoria’s Environmental Public Health Unit and attended by staff from Victoria’s Department of Health and Human Services (DHHS), EPA, and the Department of Environment, Land, Water, and Planning (DELWP). The workshops were useful in:

- formulating objectives for EPA Victoria’s environmental health tracking and providing ideas for its conceptual framework design process;
- reviewing available data sources for the EHTS;
- identifying a suitable conceptual framework for EPA Victoria’s EHTS; and
- conducting a preliminary case study within the context of the conceptual framework.

## 3. Available Conceptual Frameworks for Environmental Health Tracking

A conceptual framework for environmental health tracking provides an organized approach that helps visualize, collate, and analyze issues related to actual or predicted environmental health relationships [6,7]. In addition, conceptual frameworks can connect individual monitoring programs and support development of new indicators, policies, and programmes [8].

A literature search was conducted on conceptual frameworks for environment and health reporting globally, and several studies were identified that conducted a review on conceptual frameworks used for reporting environmental and health issues [6,7,9,10,11,12]. Thacker et al. [5] conceptual framework, Driving Force-Pressure-State Exposure-Effect-Action (DPSEEA) [13], Driving Force-Pressure-State-Impact-Response (DPSIR) [14], and Pressure-State-Response (PSR) [15] were the most common conceptual frameworks used in reporting environmental and health issues, and these were assessed for potential suitability in Victoria. 

## 4. Choosing a Conceptual Framework

A conceptual framework is required to accurately and precisely identify relationships between sources that influence change in the environment, and health impacts that can be linked to these causes support effective intervention. It is essential to understand the components of conceptual frameworks to determine which one would be most suitable for EPA Victoria EHTS. The components of the four conceptual frameworks are listed in Table 1.

In addition to understanding the components of the four conceptual frameworks, it is essential that a proposed conceptual framework enables the purposes and objectives of the environmental health tracking network to be achieved. A list of criteria for selecting a suitable conceptual framework for reporting environmental health issues is shown in Table 2. 

## 5. Availability of Data

The decision of including data into the tracking network will need to be based on relevance of the data, data quality, and compatibility of available data. Also, it would involve exploring how new and existing environmental, exposure, and adverse health effects data will be used [16] in the EHTS. EPA has identified potential collaborative stakeholders, such as DHHS Victoria, DELWP Victoria, Bureau of Meteorology (BOM) Australia, Australian Bureau of Statistics (ABS), Commonwealth Scientific and Industrial Research Organisation (CSIRO), and other state government agencies as well as universities that can provide relevant data for the EHTS. The type of relevant data held by these organisations could include environmental data such as environmental monitoring and modeling of air and water quality, land use data, and health data from hospital emergency departments and ambulance services. However, exposure data (such as direct measurements of individual exposure from personal sampling) are not currently available for ongoing tracking. Linking environmental exposure and health effects back to their determining factors is important for the EHTS; however, the unavailability of exposure data at this point in time was taken into account when designing the conceptual framework.

## 6. Proposed Conceptual Framework for EPA’s Environmental Health Tracking

An important consideration for EPA’s conceptual framework is that it needs to be able to link changes in environmental conditions and health impacts to its determining factors so that effective action can be taken to reduce or eliminate risks of adverse health effects through interventions (Table 1). Based on the information provided in the studies that were reviewed related to conceptual frameworks for environment and health reporting, DPSEEA and Thacker et al. [5] methods were the two conceptual frameworks that could link environmental exposure to health effects. However, as shown in Table 1, conceptual framework of Thacker et al. [5] did not have an action component, meaning there were no intervention points along hazard, exposure, and health surveillance components or actions to minimize or mitigate risk of adverse health effects. On the other hand, the DPSEEA conceptual framework could link adverse human health effects with environmental exposure to action, making it possible for interventions to target either environmental exposure and adverse health effects, or both, as well as their determinants throughout the cause-effect chain (Table 2) [17]. Another important aspect of the DPSEEA conceptual framework was that it was flexible and could be modified depending on environmental health surveillance requirements [7,11,17]. This is important, as EPA’s environmental health tracking requirements are expected to change over time. 

Although it was noteworthy that DPSIR had an impact component, exposure was not a listed component, and it was not considered in the association between state (changes in environmental condition) and impact (effects on human health). Also, DPSIR was criticized for providing a static representation of the environment and ignoring significant interactions between components [7,11,12]. This was a limitation, as the DPSIR conceptual framework could not be used to investigate relationships between environmental exposure to contaminants and possible health effects for specific cases. 

**Figure 1:**
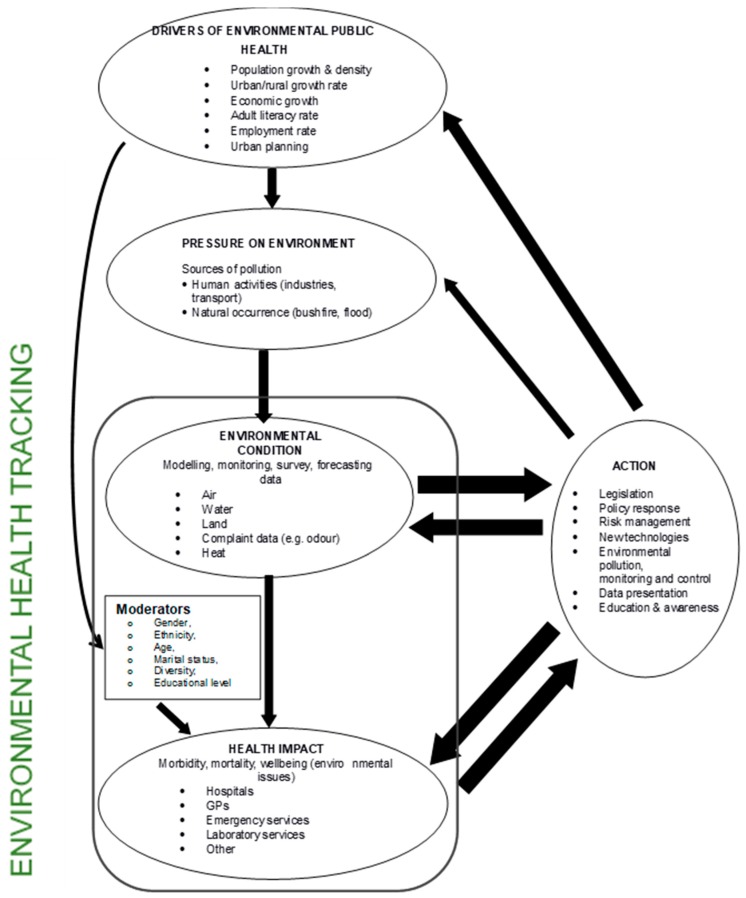
Proposed conceptual framework for EPA’s environmental health tracking.

In conclusion, the DPSEEA conceptual framework was the most suitable for EPA, as it had all the required components and criteria for environmental health tracking in Victoria (Table 1 and Table 2). Therefore, the DPSEEA conceptual framework was selected and modified to meet EPA’s environmental health tracking objectives by removing exposure and effect (Figure 1).

## 7. EPA Victoria’s Drivers-Pressure-Environmental Condition-Health Impact-Action (DPEHA) Conceptual Framework

The DPSEEA was modified to “Drivers–Pressure–Environmental Condition–Health Impact–Action” (DPEHA) because of the unavailability of exposure data from personal monitoring or biomonitoring. However, DPEHA can use proxy indicators (for exposure), such as ground-level concentrations of pollution, combined with population information such as population density. The EHTS will continue to improve and change over time by adding new data, and in the future, when exposure data may become available, DPEHA can be modified to include an exposure component that is consistent with DPSEEA. Some of the benefits of developing DPEHA are to organize indicators in a causal chain, support the development of environmental health indicators, and help develop the technical architectural framework for the EHTS. As defined by Briggs et al. [18] “*environmental health indicator is an expression of links between environment and health, targeted at a specific policy issue or management concern and presented in a form which facilitates interpretation for effective decision-making*”.

As shown in Figure 1, environmental condition and health impact are important components of the environmental health conceptual framework when describing the cause and effect relationships between environment and health in Victoria. The DPEHA conceptual framework provides an organized approach to describe ways in which drivers through human activities exert pressures on the environment. As a result, the pressure changes the environmental conditions, which in turn produces acute and chronic adverse health impacts through various exposure pathways for individuals, groups, and communities. Figure 1 also shows how actions, such as environmental and economic policies, can control and manage efforts that would lead to prevention of harmful environmental conditions that could adversely impact human health. The arrows that start at action and link to the other components, such as health impacts and pressure on the environment, indicate the influence of action along the cause–effect chain; the thicker the arrow, the stronger the influence of the action on the component. The rationale for having a stronger action (thicker arrow) on environmental condition and health impact components is that actions will ultimately affect the drivers as well as immediate pressures on the environment through economic and environmental policies and programs developed by decision makers in Victoria. Policies that reduce or mitigate environmental exposure may result in improved environmental condition by effectively managing and controlling the release of contaminants to the environment or by limiting the interaction of people with certain areas and, therefore, avoiding being exposed. The benefit of the DPEHA is that associations between change in environmental conditions and health outcomes can still be made without an exposure component. Also, useful additions to the DPEHA conceptual framework are moderators for linking drivers to change in environmental conditions and subsequent health impacts. These moderators are “qualitative or quantitative factors such as gender, ethnicity, age, marital status, educational level, housing, income and poverty status, that specifies when or to whom something may happen” [19]. The moderators will aid in providing information on diversity within a population. Also, the addition of moderators accounts for population vulnerability, which is useful in generating knowledge on spatial and temporal patterns of health, acute and chronic adverse health effects, as well as socioeconomic and demographic factors.

The DPEHA conceptual framework (Figure 1) was tested by using it to examine some potential scenarios as discussed in the next section.

## 8. Potential Application of the Environmental Health Tracking System

Two case studies of environmental health issues were selected in applying the DPEHA conceptual framework for visualizing and evaluating environmental health links in the EHTS to:

- provide scientific evidence for EPA Victoria for improved public health and the environment in the state;
- provide information for Victorians in making informed decisions on things affecting their health; and
- respond in a timely and effective way to community concerns on environmental health-related issues.

Scenario 1: understanding the spatial distribution of the health burden of noise from road traffic. 

**Figure 2:**
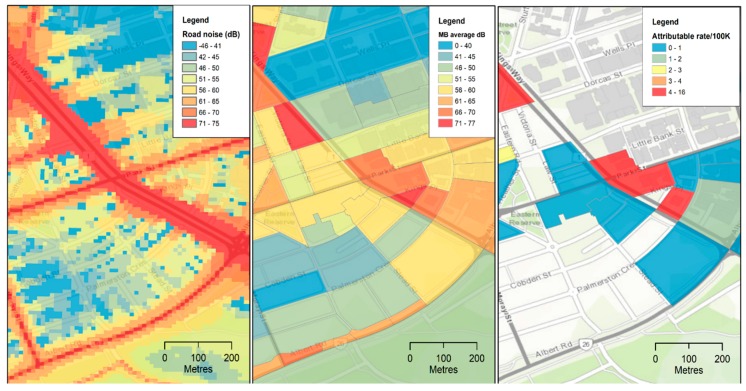
Maps showing road traffic noise exposures and expected rates of premature attributable cardiovascular deaths (per 100,000 people) in selected ABS mesh blocks of Melbourne generated using the interactive mapping application.

This project was an opportunity to demonstrate the concept of Victorian environmental health tracking in investigating the health burden of noise from road traffic in Melbourne using available data from noise mapping studies [20,21] and the ABS Census [22]. Figure 2 depicts the preliminary results from the project, with maps showing noise levels modeled at a 10 m × 10 m grid, averaged noise level per mesh block (geographical area for which data were provided by the Australian Bureau of Statistics—generally consisting of between 30 and 60 residences), and the expected rate of ischemic heart disease deaths attributable to the estimated potential exposure levels in each small area population subgroup. This mapping tool will be useful for EPA and other stakeholders in evaluating the burden of disease from road traffic noise to provide scientific evidence that guides public health decision makers in Victoria. The mapping application tool will be available on request. It will ultimately be accessed via the internet as a risk assessment tool for epidemiologists and risk assessors to support public health. This will help to identify areas and/or populations that may be impacted by noise from road traffic. 

Scenario 2: Public health investigations of anthropogenic PM_2.5_ and exacerbation of respiratory-related illnesses

**Figure 3:**
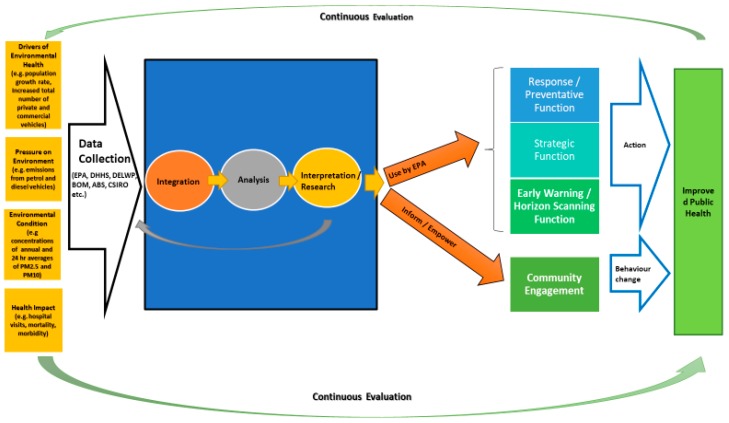
Application of Driving Force-Pressure-Environmental Condition-Health Impact-Action (DPEHA) conceptual framework showing connections amongst the various components including functionalities of environmental health tracking in investigating burden of respiratory illnesses associated with vehicle emissions of PM_2.5_ and PM_10_.

This scenario was a hypothetical example of how the DPEHA conceptual framework could be applied in estimating the burden of respiratory illnesses associated with anthropogenic PM_2.5_ (as shown in Figure 3). Common anthropogenic PM_2.5_ sources included emissions from road vehicles, planned burns, and wood heaters for heating. This scenario made use of the data in Table 3.

As shown in Figure 3, the DPEHA not only provided a platform for collecting and integrating environment and health data, and data from other sources, into the EHTS, it could also be useful to better understand the links between environmental health problems and their causes through analysis and interpretation of the data. Outcomes from analysis of the data will provide scientific information for decision makers to drive policies such as measures to mitigate or minimize emissions of PM_2.5_ associated with road traffic and public awareness. The possible action(s) will result in improved public health across Victoria. 

## 9. Conclusions

The DPEHA conceptual framework has been developed for EPA’s environmental health tracking system from DPSEEA’s conceptual framework. DPEHA’s conceptual framework provides essential features that could link changes in the environment and associated adverse health effects, which allows for effective interventions that align with the purpose of this tracking. DPEHA also provides an organized approach that supports the visualization, collation, and analysis of environmental and health relationships in tracking. This environmental health tracking system will be used to investigate and track (temporally and spatially) major environmental public health issues in Victoria as demonstrated in the case studies here. It will also allow an evidence-based approach to develop effective interventions to mitigate and prevent associated health risks.

## Figures and Tables

**Table 1:** Components of some conceptual frameworks.

Conceptual Framework	Required Attributes for Environment Protection Authority (EPA)’s Conceptual Framework					
	Driving Force	Pressure	State/Hazard Surveillance	Exposure/Exposure Surveillance	Effect/Impact/Health Surveillance	Action/Response
Thacker et al. [5]			X	X	X	
PSR * [15]		X	X			X
DPSIR ** [14]	X	X			X	X
DPSEEA *** [13]	X	X	X	X	X	X

* PSR—Pressure, State, and Response. ** DPSIR—Driving Force, Pressure, State, Impact, and Response. *** DPSEEA—Driving Force, Pressures, State, Exposure, Effect, and Action.

**Table 2:** EPA’s criteria for selecting a conceptual framework.

Conceptua Framework	Criteria for Developing EPA’s Conceptual Framework						
	Use Existing Ongoing Monitoring Data	Integrate Existing Data and New Data	Be Used in Developing Environmental Health Indicators	Linkage of Environmental Exposure and Health Effects Back to Their Determining Factors	Link Environmental Health Tracking to Public Health Action	Identify Intervention Points along Environmental Health Causal Component	Measure Impacts of Actions Taken to Improve Health
Thacker et al. [5]	X	X	X	X			
PSR * [15]	X	X					
DPSIR ** [14]	X	X	X			X	X
DPSEEA *** [13]	X	X	X	X	X	X	X

* PSR—Pressure, State, and Response. ** DPSIR—Driving Force, Pressure, State, Impact, and Response. *** DPSEEA—Driving Force, Pressures, State, Exposure, Effect, and Action.

**Table 3:** Application of the DPEHA conceptual framework to anthropogenic PM_2.5_ emissions.

DPEHA Conceptual Aspect	Data Inputs
Driving force	Population increase in Victoria
	Increased demand for road networks
	Increased total number of private and commercial vehicles (cars, vans, trucks, and buses)
	Increase in ownership of petrol and diesel vehicles
Pressure	Average age of vehicles (older vehicles may release more PM_2.5_ and PM_10_ than newer vehicles)
	Busy roads
	Emissions from petrol and diesel vehicles
	Cars not maintained well could produce more emissions
Environmental Conditions	Monitoring and modeled concentrations of ambient PM_2.5_ and PM_10_ from car emissions collected over time
Health Impact	Premature deaths and hospital admissions for respiratory and respiratory conditions
	Estimated disability-adjusted life years lost attributable to PM_2.5_ exposure
Action	Policies to improve air quality
	Use of available technologies and controls to reduce emissions from road vehicles
	Public awareness campaign
	Community engagement through educational programs
